# Genetic Polymorphism Reveals *FAT3* Gene Associations with Wool Traits in Subo Merino Sheep

**DOI:** 10.3390/ani15172534

**Published:** 2025-08-28

**Authors:** Asma Anwar, Gvlnigar Amar, Wangsheng Zhao, Wenna Liu, Shengchao Ma, Sen Tang, Cuiling Wu, Xuefeng Fu

**Affiliations:** 1 Xinjiang Key Laboratory of Special Species Conservation and Regulatory Biology, College of Life Sciences, Xinjiang Normal University, Urumqi 830017, China; asma247462@163.com (A.A.);; 2 Xinjiang Academy of Animal Husbandry Sciences, Urumqi 830000, China; 3 College of Life Sciences and Engineering, Southwest University of Science and Technology, Mianyang 621000, China

**Keywords:** Subo Merino sheep, *FAT3* gene, polymorphism, wool traits

## Abstract

In this study, 944 Subo Merino sheep, a high-quality fine wool breed, were selected as research subjects. The SNP typing of the *FAT3* gene was performed using the Fluidigm BiomarkTM HD system, and 11 missense mutation sites were identified. The analysis of population polymorphism of single-nucleotide polymorphisms was conducted. It is noteworthy that a substantial strong linkage disequilibrium was identified between SNP 5 and SNP 6 (*r*^2^ > 0.8). The association between SNPs of the *FAT3* gene and wool traits showed that multiple SNPs were significantly correlated with several different wool traits (*p* < 0.05). Furthermore, the investigation delved into the impact of the *FAT3* gene on wool fiber through the utilization of quantitative polymerase chain reaction (qPCR), which yielded findings that this gene was notably expressed in fine wool fiber (FW) (*p* < 0.001). To predict the subcellular localization and protein transmembrane structure of FAT3, we employed the PSORT II Prediction and TMHMM online software. It was determined that the protein contains a transmembrane domain. This study provides molecular markers for the improvement of the selection and breeding of ultrafine-wool sheep and offers experimental evidence for accelerating the genetic breeding of sheep.

## Simple Summary

This study focuses on the wool quality of Subo Merino sheep, which are known for their fine and soft wool. However, breeding even finer wool remains a challenge. Researchers collected blood and wool samples from 944 sheep and examined the *FAT3* gene, identifying 11 mutated sites. Ten of these mutations were significantly linked to key traits such as fiber diameter, length, and uniformity. Experiments also showed that the *FAT3* gene was more active in fine wool than in ultrafine wool. Additionally, the FAT3 protein was found mainly in the endoplasmic reticulum and nucleus of cells and has a transmembrane structure. These findings provide a scientific basis for selecting breeding stock with finer wool through genetic testing, helping to accelerate the breeding of high-quality fine-wool sheep, ultimately improving the market competitiveness of wool products and benefiting both farmers and consumers.

## 1. Introduction

The quality and yield of wool are among the most important economic traits of sheep and directly determine the financial benefits of animal husbandry [1]. High-quality wool significantly increases the added value of products, thereby enhancing the market competitiveness of animal husbandry [2]. The Subo Merino sheep is the first fine-wool breed of sheep in China bred for worsted use. It was bred using Xinji fine-wool sheep and Australian Merino superfine breed rams as the male parents and Chinese merino sheep, Xinji fine-wool sheep, and Aohan fine-wool sheep as the female parents. The wool fiber diameter is 17.0–19.0 µm, making it the first fine-wool sheep bred for worsted use in China [3]. The wool fiber of Subo Merino sheep is 19.1–23.0 µm, which is higher than that of Chinese Merino and Xinji fine-wool sheep. The quality of the wool is among the best in Chinese fine-wool sheep. Compared to Australian Merino superfine sheep, Subo Merino sheep have a higher wool yield and longer wool length at the same level of fineness (80). These characteristics make Subo Merino sheep an ideal model for studying the molecular regulation of wool fineness. Despite the volatility of the global wool market, China remains one of the world’s largest importers of wool. Improving the quality of domestic fine wool is important for reducing dependence on imports. However, enhancing wool traits genetically has always been a core challenge in sheep breeding. While traditional breeding methods have improved wool quality to some extent, it is difficult to optimize multiple traits simultaneously. Excessive selection of a single trait may lead to antagonistic effects between energy metabolism and hair follicle development [4]. Due to the rapid development of molecular biology technology, gene polymorphism research has become an important means of analyzing the genetic basis of wool traits. Recently, several studies on complex sheep traits have been reported. For the first time, Wang et al. [5] found nine genes significantly related to wool fiber diameter, three genes related to fiber diameter coefficient of variation, two genes related to fiber dispersion, and fifteen genes considerably related to wool bending number in 765 individuals of six strains of Chinese Merino sheep (Junken type). Gene polymorphism analysis can deeply explore functional genes and molecular markers related to wool traits, providing a new theoretical basis and technical support for molecular marker-assisted selection [6]. Bai et al. [7] revealed the significant association between *KRTAP19-5* gene variation and fine wool fiber curvature by analyzing the gene polymorphism of 241 Chinese Tan sheep. Based on GWAS, Zhao et al. [8] screened eight SNPs related to fiber diameter, fiber diameter correlation coefficient, coefficient of variation, wool length, shearing amount, net wool rate, short fiber strength, and short fiber elongation from 460 fine-wool sheep. In addition, Zhao et al. [9] found that five SNPs in the *FGF5* gene significantly affected the wool traits and growth performance of fine-wool sheep. Wang et al. [10] revealed the relationship between the *PTPN3* gene SNP, wool yield, and growth traits in dual-purpose sheep. This indicates that gene polymorphism analysis can deeply explore functional genes and molecular markers related to wool traits. These results further confirm the effectiveness of gene polymorphism analysis in identifying functional genes and molecular markers related to wool traits and provide a new theoretical basis and technical support for molecular marker-assisted selection.

In recent years, genes related to fat metabolism have received extensive attention due to their dual roles in regulating the hair follicle cycle and lipid synthesis. In skin tissue, the expression of different genes may affect the proliferation and differentiation of keratinocytes in the hair follicle cycle, thereby altering fiber diameter and bending characteristics [11]. At the same time, genes that mediate lipid metabolic pathways may provide substrates for lanolin synthesis, thereby affecting fiber elasticity and fineness [12]. The *FAT3* gene, a member of the non-classical cadherin family, plays a key role in cell adhesion, signal transduction, and tissue morphogenesis [13]. Studies have shown that *FAT3* regulates the proliferation and differentiation of neural progenitor cells by interacting with the Hippo signaling pathway [14]. Currently, there is a lack of systematic research on the polymorphism distribution of the *FAT3* gene in Subo Merino sheep, particularly regarding its association with wool traits. The genetic variation characteristics of fine- and ultrafine-wool sheep have not been fully analyzed. Therefore, we utilized genetic polymorphism analysis techniques to conduct a systematic analysis of the *FAT3* gene in Subo Merino sheep, providing a solid theoretical and practical foundation for the genetic breeding of ultrafine-wool sheep. Through high-throughput SNP genotyping, this research thoroughly revealed the polymorphic characteristics of the *FAT3* gene and its associations with wool fiber traits. Furthermore, the expression differences of this gene in different types of wool fibers were validated using qPCR technology. These research findings not only offer new insights into the molecular regulatory mechanisms of wool fiber traits but also provide important scientific evidence for molecular marker-assisted selection strategies, contributing to the advancement of genetic improvement in ultrafine-wool sheep.

## 2. Materials and Methods

### 2.1. Sample Collection and Wool Phenotype Determination

A total of 944 one-year-old Subo Merino ewes were obtained from two farms: Gongnais (*n* = 473) in Yili Prefecture, Xinjiang Uygur Autonomous Region, and Baicheng (*n* = 471) in Aksu City. Venous blood from the experimental sheep was collected in 5 mL anticoagulant tubes and stored in a refrigerator at −20 °C for subsequent single-nucleotide polymorphism (SNP) typing. Wool samples were collected 10 cm from the posterior edge of the scapula, above the midline of the left side of the sheep. Greasy fleece weight (GFW), live weight before shearing (LWBS), and live weight after shearing (LWAS) were measured at the same time. The wool fiber diameter was measured using an optical fiber diameter analyzer (OFDA-2000BT, BSC Electronics, Perth, Australia) in the laboratory. The mean fiber diameter (MFD), fiber diameter standard deviation (FDSD), fiber diameter coefficient of variation (CVFD), staple length (SL), fineness count (FC), hair length (HL), and crimp number (CN) were measured. We used SPSS 29.0.2.0 software to conduct a comprehensive descriptive statistical analysis of data related to wool traits to understand the basic characteristics and distribution of wool. According to the average fiber diameter, sheep were divided into the FW group and the UFW group; 10 sheep were randomly selected in each group. Although qPCR validation was limited to 10 animals per group, the large effect size (Cohen’s d > 5.0) and both groups exhibited low within-group variability (FW: CV = 4.6%; UFW: CV = 2.4%), suggesting this sample size was sufficient to detect biologically meaningful differences. Skin samples were collected and stored in liquid nitrogen for subsequent RNA extraction from skin tissue. To show the difference between fine and ultrafine wool more intuitively, GraphPad Prism 8.0.2 software was used for mapping.

### 2.2. SNP Genotyping of the FAT3 Gene

In this study, high-throughput single-nucleotide polymorphism (SNP) genotyping was performed on 944 individuals using the Fluidigm Biomark™ HD System (Biomark™ HD, San Francisco, CA, USA). This system is a high-throughput genotyping platform based on microfluidic technology and real-time quantitative PCR (qPCR). It combines these technologies to efficiently and accurately complete reactions. It is primarily used to detect single-nucleotide polymorphisms (SNPs) in known genes. The information on the *FAT3* gene primer pair is shown in Table 1.

### 2.3. Population Polymorphism Analysis

The R 4.4.1 project was used to calculate the genotype frequency, number of minor allele frequencies (MAF), effective alleles (Ne), homozygosity (Ho), heterozygosity (He), and polymorphism information content (PIC). The Hardy–Weinberg equilibrium and linkage disequilibrium were analyzed using HaploView 4.2 software.

### 2.4. Correlation Analysis Between SNPs of the FAT3 Gene and Wool Phenotypic Traits

We used the SAS 9.4 program and the least squares variance analysis method to analyze the correlation between different genotypes of SNPs and wool traits. We used different genotypes of SNPs and different sheep farms as fixed effects. We estimated the least squares mean ± standard error of wool traits. The linear model was as follows:

Y_ick_ = µ + G_i_ + F_c_ + e_ick_ (1)

In the formula, Y_ick_ is the phenotypic value of an individual fine-wool sheep; µ is the group mean; G_i_ is the SNP genotype effect; F_c_ is the field effect, to correct for systematic differences in wool traits arising from variations in husbandry practices, climatic conditions, and measurement protocols across different test stations; and e_ick_ is the random error.

To account for multiple testing across 11 SNPs and 10 traits, we applied Benjamini–Hochberg false discovery rate (FDR) correction. Associations with FDR-adjusted q-values < 0.05 were considered statistically significant.

### 2.5. Quantitative Expression Analysis and Protein Structure Prediction

The cutaneous tissues RNA extraction kit (Tiangen Biochemical Technology (Beijing) Co., Ltd., Beijing, China) was used to extract cutaneous tissue RNA from the FW and UFW groups of Subo Merino sheep. According to the NCBI database, the *FAT3* target gene and the GAPDH exon sequence were obtained as the internal reference gene, and primers were designed using Primer Premier 5.0 (Table 1). The primers were synthesized by Youkang Biological (Urumqi, China) Co., Ltd. The PCR reaction system consisted of the following components: PCR mixture (10 µL), upstream and downstream primers (0.5 µL each), DNA template (1.0 µL), and distilled water (20 µL). The established PCR system was used to perform quantitative PCR on a Linegene 9660 quantitative PCR detection system (Bori Technology, Hangzhou, China). The reaction conditions were as follows: pre-denaturation at 95 °C for 3 min, denaturation at 94 °C for 30 s, annealing at 60 °C for 34 s, 45 cycles of this process, a dissolution curve analysis, and preservation at 4 °C. The mRNA expression changes of the *FAT3* gene in different fine wools were visualized using GraphPad Prism software.

The subcellular localization of sheep FAT3 protein was predicted using PSORT II Prediction online software (https://www.genscript.com/psort.html, accessed on 25 August 2025), and the transmembrane structure region of the protein was analyzed using TMHMM online software.

## 3. Results

### 3.1. Descriptive Statistics of Traits

Descriptive statistics on the wool traits of Subo Merino were calculated using SAS 9.4 software. The basic statistics are shown in Table 2. The average and range of each trait align with the objective facts. The small standard deviation indicates a relatively dense distribution of variables near the average. The coefficient of variation reflects the degree of variation of each trait.

### 3.2. Determine the SNPs and Genetic Polymorphism Analysis of FAT3 Gene

In this study, high-throughput SNP genotyping was performed on 944 individuals using the Fluidigm Biomark™ HD System (Biomark™ HD, USA). After sequence alignment analysis, 11 missense mutation SNPs were detected (Table 3). Missense mutations affect the structure and function of the proteins they encode. Therefore, this experiment focuses on verifying the results of the missense mutation sites.

This investigation analyzed the genotype and minor allele frequencies (MAF) of 11 SNP markers of the *FAT3* gene (Table 4). The results showed that the minor allele distributions of SNPs 2 and 6 (MAF = 0.460 and 0.477, respectively) were balanced (MAF was close to 0.5), suggesting high polymorphism. SNP 7 (MAF = 0.003) may be a monomorphic locus due to the extreme rarity of minor alleles (only four heterozygotes). The allelic distribution of SNPs 3 and 11 (MAF = 0.293 and 0.401, respectively) is moderate and suitable for population genetics research. Regarding the genotype distribution, the proportion of homozygous rare genotypes of SNP 9 (AA = 8) and SNP 10 (CC = 28) was extremely low (<1%), suggesting potential selection pressure or a population bottleneck effect. Conversely, the proportion of heterozygotes for SNPs 5 (TC = 303) and 11 (GA = 409) was significantly higher than that of other loci, which may reflect heterosis or population admixture.

This research analyzed the genetic diversity of 11 single-nucleotide polymorphism (SNP) markers of the *FAT3* gene (Table 4). The results showed that the polymorphism information content (PIC) of SNPs 2 and 6 (PIC = 0.651 and 0.633, respectively) was significantly higher than that of the other loci (PIC > 0.6), indicating their potential as high-information markers. The expected heterozygosity of SNP 7 was extremely low and significantly deviated from Hardy–Weinberg equilibrium. The heterozygosity distribution showed that the homozygosity (Ho) of SNPs 3 and 8 was significantly lower than the heterozygosity (He); the heterozygosity of SNP 5 was consistent with the Hardy–Weinberg equilibrium, indicating its genetic stability. The remaining SNPs’ genetic parameters were within a reasonable range.

### 3.3. Linkage Disequilibrium Analysis

**Figure 1:**
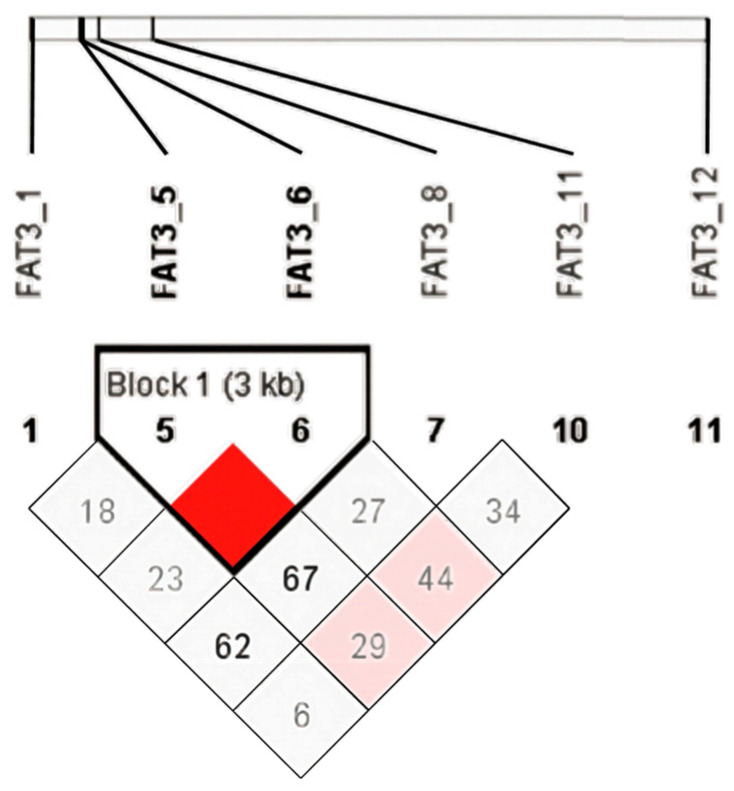
Haplotype block diagram.

This study analyzed linkage disequilibrium between 11 single-nucleotide polymorphisms (SNPs) in the *FAT3* gene region (Figure 1). In the figure, different colors represent the intensity of the r^2^ value: red indicates strong linkage disequilibrium (r^2^ > 0.8), pink indicates moderate linkage disequilibrium (0.5 ≤ r^2^ ≤ 0.8), and white indicates weak linkage disequilibrium (r^2^ ≤ 0.5). Notably, there is a significant strong linkage disequilibrium between SNPs 5 and 6, suggesting that they are located in close proximity within the same gene. Additionally, Block 1, marked in the figure, shows a set of highly linked SNPs.

### 3.4. The Association Analysis of the FAT3 Gene with Wool Traits

Our study analyzed the association between the *FAT3* gene SNPs and different wool traits (Table 5). The results showed a significant difference in MFD between individuals with AA and CC genotypes compared with AC genotypes, in SNP 1 (*p* < 0.05). There was also a significant difference in FDSD between individuals with the AC and CC genotypes compared to the AA genotype (*p* < 0.001). There was a significant difference in CVFD (*p* < 0.05). There was a significant difference in LWBS between individuals with AA and AC genotypes (*p* < 0.05). In SNP 2, significant differences were observed in MFD and FDSD between individuals with GA and GG genotypes and those with AA genotypes (*p* < 0.05), in FC and HL between individuals with GA and GG genotypes (*p* < 0.05), and in CN between individuals with AA and GA genotypes (*p* < 0.05). There were significant differences in MFD (*p* < 0.05) and FDSD (*p* < 0.001) between CC and TC genotypes in SNP 3. A significant difference in FC was observed between the CC and GG genotypes, as well as the GC genotypes, at SNP 4 (*p* < 0.05). There was a significant difference in LWBS between the CC and TC genotypes and the TT genotype in SNP 5 (*p* < 0.05). In SNP 6, there were significant differences in MFD and SL between the CC and CT genotypes and the TT genotype (*p* < 0.05), as well as significant differences in FC between the CC and TT genotypes (*p* < 0.05). The MFD between the CC and TT genotypes in SNP 8 was significant (*p* < 0.001). In SNP 9, there was a significant difference in HL between the AA and AG genotypes and the GG genotype (*p* < 0.05). In SNP 10, there were significant differences in HL between the TC and TT genotypes (*p* < 0.05) and in LWBS between the CC, TC, and TT genotypes (*p* < 0.05). In SNP 11, there were significant differences in MFD between the GA and GG genotypes (*p* < 0.05) and in HL between the AA and GG genotypes (*p* < 0.05). There was no significant effect on individual hair traits between different genotypes of SNP 7.

### 3.5. Comparative Analysis of FAT3 Gene Expression in Ultrafine Wool and Fine Wool Fibers

**Figure 2:**
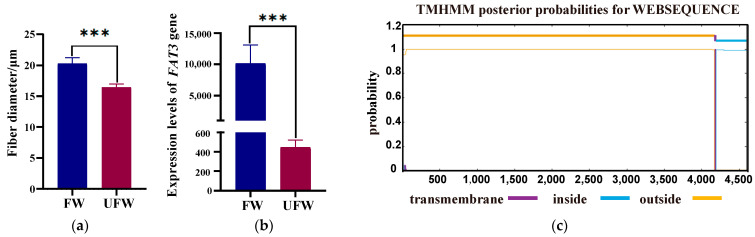
(**a**) The difference between ultrafine and fine wool fibers. (**b**) The mRNA expression of the *FAT3* gene between the 2 groups. *** means extremely significant difference (*p* < 0.001). (**c**) The transmembrane structure of sheep *FAT3* gene.

After measuring the MFD of 944 Subo Merino sheep, we selected 10 representative sheep according to their fiber diameter extremes for subsequent verification and divided them into two groups. Specifically, we classified the fiber diameter of 19.7–22.7 µm as the FW group, and the fiber diameter of 15.3–16.7 µm as the UFW group. The results showed that the diameter of ultrafine wool fibers was significantly smaller than that of fine wool fibers (*p* < 0.001) (Figure 2a). We analyzed the mRNA expression of the *FAT3* gene in the two types of wool fibers by qPCR (Figure 2b). The results showed that *FAT3* gene expression in fine wool was significantly higher than in ultrafine wool (*p* < 0.001).

### 3.6. Protein Transmembrane Structure Analysis

The subcellular localization of the sheep FAT3 protein was predicted using the PSORT II prediction software. The results showed that FAT3 protein distribution in the endoplasmic reticulum and nucleus accounted for 33.3%. The proportion of the secretory system, vacuoles, and the Golgi apparatus was 11.1%. The transmembrane helix of the FAT3 protein was predicted using the online TMHMM2.0 software (Figure 2c). The final result showed that the FAT3 protein is 4602 amino acids long and has one predicted transmembrane helix (TM). The number of amino acids in the transmembrane helix region was 21.85362, and the expected number of predicted transmembrane helix regions was 1.15407. The total N-terminal entry probability is 0.04731. Figure 2c shows the predicted number of transmembrane helices and the expected number of amino acids in the transmembrane helices. The lower part shows the posterior probability of the transmembrane helices. The figure shows that the purple line represents the transmembrane region, the yellow line represents the outer region, and the blue line represents the inner region. As can be seen, the transmembrane region is concentrated primarily in positions 4170 to 4189 of the sequence. The probability of this region being a transmembrane helix is close to 1. None of the missense variants reside within the predicted transmembrane segment, suggesting no structural impact on membrane anchoring.

## 4. Discussion

### 4.1. Effects of Missense Mutation and Expression Difference of the FAT3 Gene on Wool Traits

This study has a large sample size of 944 sheep individuals, which provides sufficient statistical support for the research results. Based on this, we successfully identified 11 missense mutation SNPs in the *FAT3* gene. These missense mutations are biologically significant because they alter the amino acid sequence, affecting the structure and function of the encoded protein [15]. Missense mutations are generally considered a type of functional variation that can significantly affect wool fiber traits [16]. For example, Sulayman et al. [17] found that missense mutations in keratin genes (such as *KRT31*, *KRT36*, *KRT38*, and *KRT85*) were significantly associated with traits such as wool fiber diameter, fineness score, and curl number. Another study found that missense mutations in the keratin gene *KRT25* (such as p.Leu317Pro) can destroy the α-helix structure of keratin and affect its heterodimerization with other keratins (such as *KRT71*), resulting in abnormal hair structure [18]. The discovery of these missense mutation single-nucleotide polymorphisms (SNPs) suggests that these *FAT3* gene mutation sites may be closely related to wool fiber fineness traits. Fineness is one of the most important indicators of wool quality, and gene variation often regulates wool fiber growth and development by affecting protein expression and function [19]. Missense mutations in these key sites may alter their regulatory effect on the fineness of wool fibers, resulting in significant differences in the diameter of fine and ultrafine fibers.

This study measured the diameter of ultrafine and fine wool fibers and analyzed the expression of the *FAT3* gene in these two types of fibers. The results revealed a significant correlation between *FAT3* gene expression and fiber diameter. The results showed that the diameter of ultrafine wool fibers was significantly smaller than that of fine wool fibers (*p* < 0.001), while *FAT3* gene expression in fine wool fibers was significantly higher than in ultrafine wool fibers (*p* < 0.001). These results suggest that *FAT3* gene expression levels may be closely related to wool fiber diameter and that differences in *FAT3* gene expression may regulate fiber diameter by affecting cell metabolism or the fiber formation process. The smaller the diameter, the softer and more comfortable the wool [20]. Therefore, understanding the relationship between *FAT3* gene expression and fiber diameter is significant for improving wool quality. The protein encoded by the *FAT3* gene may be involved in cell metabolism, cytoskeleton formation, or cell signaling, thereby affecting the growth and development of wool fibers. In fine wool, higher expression of the *FAT3* gene may promote the growth of wool fibers, resulting in a larger fiber diameter. Conversely, in ultrafine wool, lower *FAT3* gene expression may limit fiber growth, resulting in finer fibers.

### 4.2. Population Genetics Analysis of the FAT3 Gene

This study analyzed the genetic polymorphism of 11 single-nucleotide polymorphism (SNP) markers of the *FAT3* gene in detail, including gene and genotype frequencies, as well as population genetic parameters. Genotypic frequency analysis revealed that the minor allele frequency (MAF) of SNPs 2 and 6 was close to 0.5, suggesting high polymorphism at these loci. This high polymorphism may reflect neutral or balanced selection of these loci in the population, allowing the two alleles to persist over time. Highly polymorphic loci typically exhibit high genetic diversity within a population. This diversity not only helps maintain genetic stability within a population but may also be related to adaptive evolution [21,22]. Many genes related to the immune response (such as *MHC* genes) usually exhibit high polymorphism. For example, the MHC class II DAB gene of the yellow-billed egret is highly polymorphic [23]. The *MHC* gene is a typical adaptive molecular marker. Its high polymorphism enhances the population’s immunity, thereby maintaining its genetic stability and adaptability. This polymorphism helps populations adapt to different environmental pressures [24]. Similarly, the high polymorphic loci of the MAF3 gene may play an important role in regulating wool fiber traits, particularly in adapting to different environments and selection pressures. In contrast, the MAF of SNP 7 is extremely low (0.003). Together with the genotyping results, this site is monomorphic or has undergone strong purification in the population, resulting in a very low frequency of minor alleles. Additionally, the MAFs of SNP 3 and SNP 11 are moderate and suitable for population genetics research. These loci may confer certain selection advantages to the population or exist in a state of dynamic equilibrium. Regarding genotype distribution, the proportion of homozygous rare genotypes of SNP 9 and SNP 10 is extremely low, suggesting potential selection pressure or a population bottleneck effect. This phenomenon may be related to historical population events (such as inbreeding or selective breeding), resulting in the gradual reduction of certain genotypes [25]. On the contrary, the proportion of heterozygotes for SNP 5 and SNP 11 was significantly higher than that for other loci, which may reflect heterosis or population admixture. The heterozygous advantage is the phenomenon in which heterozygotes are more adaptable than homozygotes, a trait common in many organisms, particularly those with genes related to environmental adaptability [26,27]. These results suggest that the *FAT3* gene exhibits high polymorphism and genetic variability, as well as significant selection potential. This information is important for understanding the genetic variation and potential function of the *FAT3* gene in the Subo Merino sheep population and serves as a reference for improving different breeding strategies. The results show that, in follow-up practice, the intensity of artificial selection should increase to eliminate unfavorable individuals.

In this study, we found a strong linkage disequilibrium (*r*^2^ > 0.8) between SNPs 5 and 6, indicating that they are located in a tightly linked region of the same gene. SNP 5 (chr21:1529421, exon 18) and SNP 6 (chr21:1533151, exon 17) both reside in the coding sequence of *FAT3*, introducing missense variants p.Val→Ala and p.Thr→Met, respectively. Their strong LD (r^2^ > 0.8) indicates that haplotype-based selection targeting the combined alleles in Block 1 (Figure 1) is more efficient than single-SNP selection. Prioritizing this haplotype could yield a cumulative reduction of 0.5–0.8 µm in mean fiber diameter within one generation (see effect sizes in Table 5). This close linkage may mean that these two sites have a synergistic effect on gene regulation or function, or that they affect a biological process together [28]. Tightly linked SNPs may co-regulate gene expression and function. For instance, they could be situated in the promoter or enhancer region of the gene, influencing its transcriptional activity through a synergistic effect [29]. In addition, this close linkage may be related to the gene’s evolutionary history, and these sites may have been preserved together during evolution because they influence the gene’s function or adaptability. Block 1, marked in Figure 1, shows a set of highly linked SNPs. This block of highly linked SNPs may indicate an important synergistic effect on gene regulation or function. These SNPs may collectively affect the expression level, splicing pattern, or protein function of the *FAT3* gene.

### 4.3. Association Analysis Between SNPs of the FAT3 Gene and Wool Traits

Wool fiber diameter, length, fineness, and yield are important economic traits in the fine-wool sheep industry and the main factors affecting wool price. Breeding wool traits is important for improving the quality and yield of wool [30,31]. Environmental factors and minor polygenic factors jointly determine wool quality. Therefore, the objective of this study was to strictly control the association between *FAT3* gene polymorphism and wool traits under non-genetic factors, such as field, variety, and age. The study found that the association was significantly related to multiple important wool traits. The field effect was found to have a significant impact on the wool traits of different SNPs, suggesting that the intensity of artificial selection could be increased to improve this population. Additionally, this study revealed the potential role of these loci in forming hair characteristics by analyzing the correlation between multiple *FAT3* gene SNPs and hair traits.

In our study of wool fiber-related traits, we found that multiple genotypes (SNP 1, SNP 2, SNP 3, SNP 6, SNP 8, and SNP 11) showed significant correlations with MFD. A significant correlation with FDSD was also observed for SNPs 1, 2, and 3. Significant results for CVFD were mainly concentrated in SNPs 1 and 3. Wool fibers typically have elliptical rather than circular cross-sections. Studies have shown that higher FDSD and CVFD are associated with increased ovality [32]. Additionally, changes in FDSD and CVFD may reflect changes in fiber diameter [33]. CVFD is a relative index that measures the degree of fiber diameter variation [34], and its significance indicates that SNPs in the *FAT3* gene may regulate the stability of fiber diameter. Therefore, some variations in the *FAT3* gene may affect fiber diameter uniformity, which affects hair quality and consistency.

Taken together, the significant correlations among fiber diameter-related traits (MFD, FDSD, and CVFD) suggest that the *FAT3* gene plays an important role in hair follicle development, particularly in regulating hair fiber growth and uniformity. This regulation may be closely related to the proliferation and differentiation of cells in hair follicles, as well as the synthesis and assembly of hair proteins. In our study of wool length-related traits, we found that SL was significantly associated with SNP 6, and HL was significantly associated with SNPs 2, 10, and 11. These results suggest that certain SNPs in the *FAT3* gene exert direct or indirect regulatory effects on hair length. Specifically, a mutation in the *FAT3* gene may regulate hair length by affecting the growth cycle of hair follicles or the growth rate of hair. The growth cycle of hair follicles includes the growth, catagen, and telogen periods [35,36]. We speculate that SNPs in the *FAT3* gene may regulate hair length and growth rate by affecting the conversion of these stages.

### 4.4. The Effect of the FAT3 Protein on Wool Traits

Further bioinformatics analysis provides important clues for understanding the function of the FAT3 protein. PSORT II Prediction software predicted that the FAT3 protein is primarily distributed in the endoplasmic reticulum and nucleus, accounting for 33.3% and 33.3%, respectively. The endoplasmic reticulum is a key site for protein synthesis and folding, while the nucleus is a core region for regulating gene expression [37,38]. The distribution of the FAT3 protein in these two organelles suggests that it may regulate protein synthesis, modification, and gene expression in wool fiber cells. This functional characteristic may be closely related to the growth and development of wool fibers, particularly concerning the important economic trait of determining their thickness. Additionally, TMHMM2.0 software predicted that the FAT3 protein has a transmembrane helix and that the probability of the transmembrane region is close to 1. This indicates that the region is likely a transmembrane structure. The presence of transmembrane helices suggests that the FAT3 protein may be anchored to a cell or organelle membrane through its transmembrane structure, thereby exerting its biological function within cells. For example, transmembrane helices are involved in processes such as cell signaling, material transport, and cell-to-cell interactions, which are essential for the growth and differentiation of wool fiber cells [39,40,41]. The transmembrane property of the FAT3 protein may enable it to directly or indirectly regulate the formation of wool fibers, thereby affecting their properties.

Wool fibers are primarily composed of keratinocytes that differentiate and secrete keratin in the hair follicles to form the fibers [42]. We speculate that *FAT3* gene expression may affect keratin synthesis and secretion by regulating keratin cells’ metabolic activity or intercellular signal transduction, thereby affecting wool fiber diameter. Future research could explore the specific mechanism of the *FAT3* gene in wool fiber formation through experiments such as gene knockout or overexpression to verify its function.

## 5. Conclusions

In this study, the polymorphism of the *FAT3* gene was analyzed about wool traits in Subo Merino sheep. An investigation was conducted to determine the prevalence of genetic variation in the *FAT3* gene among Subo Merino sheep. The analysis revealed a high degree of polymorphism within this gene, with multiple single-nucleotide polymorphisms (SNPs) exhibiting a strong correlation with economically significant wool traits. These traits include measures of fiber diameter (MFD), fiber diameter skew (FDSD), coefficient of variation of fiber diameter (CVFD), staple length (SL), fiber count (FC), hair length (HL), and fiber consistency (FC). Notably, specific SNPs, namely SNP 1, SNP 2, SNP 3, SNP 6, SNP 8, and SNP 11, demonstrated a particularly strong association with the fineness and length of wool fiber. It has been posited that these sites may play an important role in the growth and development of wool fibers. The qPCR analysis revealed that the expression level of the *FAT3* gene in fine wool was significantly higher than that in ultrafine wool, suggesting that its expression level was closely related to wool fiber diameter. Furthermore, LD analysis revealed abundant genetic variations in the *FAT3* gene region, particularly the strong linkage disequilibrium between SNP 5 and SNP 6. This finding suggests that these two SNP loci may have synergistic effects on gene regulation or function and collectively influence wool fiber traits.

## Figures and Tables

**Table 1:** Primer information.

Gene	Primer (5′→3′)	
*FAT3*	F: CAGTCCCTGAGTTCCTTCCA	R: CGTATGGTGGGCACTCTTCT
*GAPDH*	F: GGTGATGCTGGTGCTGAGTA	R: CAGCAGAAGGTGCAGAGATG

**Table 2:** Descriptive statistics of traits.

Traits	N	Mean ± SD	CV (%)	Range
MFD/µm	940	17.71 ± 1.84	10.39	13.3~24.0
FDSD/µm	933	4.14 ± 0.57	13.77	3.0~6.4
CVFD/%	941	23.38 ± 2.24	9.58	17.0~30.8
SL/cm	929	88.26 ± 11.77	13.34	65~130
HL/cm	933	9.89 ± 0.95	9.61	7~14
FC	934	66.82 ± 2.52	3.77	60~80
CN	936	13.92 ± 3.04	21.60	7~25
LWBS/kg	936	33.18 ± 5.03	15.16	22~50
GFW/kg	765	3.33 ± 0.54	16.22	2.0~5.6
LWAS/kg	668	34.13 ± 4.96	14.53	22~50

**Table 3:** The SNPs information of *FAT3* gene.

SNPs	Chr ID	Location/bp	Exon	Ref	Alt	Mutation Type
SNP 1	21	1,481,091	30	A	C	missense mutations
SNP 2	21	1,495,094	26	G	A	missense mutations
SNP 3	21	1,501,253	23	T	C	missense mutations
SNP 4	21	1,501,292	23	G	C	missense mutations
SNP 5	21	1,529,421	18	T	C	missense mutations
SNP 6	21	1,533,151	17	C	T	missense mutations
SNP 7	21	1,547,134	13	C	A	missense mutations
SNP 8	21	1,547,314	13	C	T	missense mutations
SNP 9	21	1,587,685	9	C	T	missense mutations
SNP 10	21	1,599,982	7	A	G	missense mutations
SNP 11	21	2,136,457	1	T	C	missense mutations

**Table 4:** Genetic Polymorphism Analysis of the *FAT3* gene.

SNPs	Genotype	N	Freq	MAF	Ho	He	Ne	PIC	HWE
SNP 1	AA/AC/CC	68/312/424	A/C 0.237/0.614	0.279	0.33	0.57	2.31	0.564	0.3614
SNP 2	GG/GA/AA	380/220/307	A/G 0.441/0.519	0.460	0.23	0.54	2.15	**0.651**	1.2875
SNP 3	TT/TC/CC	615/42/243	C/T 0.280/0.674	0.293	**0.05**	**0.47**	1.88	0.458	4.5577
SNP 4	GG/GC/CC	695/138/89	C/G 0.158/0.809	0.170	0.15	0.32	1.47	0.390	7.7370
SNP 5	CC/TC/TT	603/303/34	C/T 0.799/0.197	0.198	0.32	0.32	1.48	0.483	0.5594
SNP 6	TT/CT/CC	200/408/239	C/T 0.469/0.428	0.477	0.43	0.60	2.48	**0.633**	0.3400
SNP 7	TT/TC/CC	1/3/939	C/T 0.996/0.003	0.003	0.03	0.01	1.01	0.009	0.0107
SNP 8	CC/CT/TT	406/108/168	C/T 0.487/0.235	0.326	**0.11**	**0.71**	3.42	0.343	1.1049
SNP 9	GG/AG/AA	505/127/8	A/G 0.076/0.602	0.112	0.13	0.63	2.71	0.179	1.0000
SNP 10	TT/TC/CC	627/289/28	C/T 0.183/0.817	0.183	0.31	0.30	1.43	0.254	0.5492
SNP 11	GG/GA/AA	355/409/170	A/G 0.397/0.593	0.401	0.43	0.49	2.00	0.365	0.0057

Note: PIC ≥ 0.5 is high polymorphism, 0.25 ≤ PIC < 0.5 is moderate polymorphism, PIC < 0.25 is low polymorphism. Significance is indicated by bold type.

**Table 5:** Correlation results of *FAT3* gene SNPs and the wool traits (least squares mean ± standard error).

SNPs	Type	MFD (µm)	FDSD (µm)	CVFD (%)	SL (cm)	HL (cm)	FC	CN	LWBS (kg)	GFW (kg)	LWAS (kg)
SNP 1	AA	**18.04 ^a^**	**4.34 ^A^**	**24.03 ^a^**	86.96	9.98	66.63	14.05	**32.28 ^a^**	3.16	32.65
	AC	**17.66 ^b^**	**4.11 ^B^**	**23.28 ^b^**	88.48	9.94	67.03	13.68	**33.49 ^b^**	3.29	32.98
	CC	**17.70 ^b^**	**4.13 ^B^**	**23.34 ^b^**	88.61	9.86	66.76	13.95	**33.06 ^ab^**	3.27	32.81
SNP 2	AA	**17.54 ^a^**	**4.07 ^a^**	23.22	88.49	**9.86 ^ab^**	**66.82 ^ab^**	**14.15 ^a^**	33.05	3.26	32.71
	GA	**17.72 ^ab^**	**4.18 ^b^**	23.57	87.44	**9.83 ^a^**	**67.15 ^a^**	**13.64 ^b^**	33.18	3.27	33.02
	GG	**17.80 ^b^**	**4.15 ^b^**	23.35	89.02	**9.98 ^b^**	**66.68 ^b^**	**13.90 ^ab^**	33.44	3.28	33.03
SNP 3	CC	**17.46 ^A^**	**4.06 ^A^**	23.26	88.25	9.88	67.06	13.97	33.27	3.29	32.79
	TC	**17.45 ^AB^**	**4.15 ^AB^**	23.78	86.44	9.80	67.16	13.61	32.47	3.11	33.29
	TT	**17.81 ^B^**	**4.17 ^B^**	23.41	88.56	9.92	66.75	13.92	33.09	3.27	32.89
SNP 4	CC	17.73	4.08	23.06	86.98	9.89	**66.40 ^a^**	13.88	32.73	3.25	32.72
	GC	17.67	4.12	23.32	88.11	9.86	**67.41 ^b^**	14.12	33.61	3.29	33.25
	GG	17.69	4.14	23.43	88.18	9.88	**66.74 ^a^**	13.86	33.07	3.26	32.82
SNP 5	CC	17.66	4.12	23.35	87.99	9.86	66.85	13.90	**33.18 ^a^**	3.25	32.84
	TC	17.80	4.18	23.47	88.94	9.96	66.77	13.95	**33.31 ^a^**	3.33	33.18
	TT	17.56	4.08	23.25	86.58	9.75	66.56	13.58	**31.75 ^b^**	3.13	32.00
SNP 6	CC	**17.79 ^A^**	4.15	23.32	**89.07 ^a^**	9.97	**66.63 ^a^**	13.69	33.44	3.29	33.12
	CT	**17.72 ^A^**	4.15	23.42	**88.68 ^a^**	9.91	**66.88 ^ab^**	13.90	33.47	3.27	32.89
	TT	**17.41 ^B^**	4.07	23.43	**86.65 ^b^**	9.88	**67.13 ^b^**	13.93	32.93	3.24	32.63
SNP 7	CC	17.71	4.14	23.38	88.28	9.89	66.83	13.90	33.17	3.27	32.91
	CT	17.38	4.27	24.64	88.86	9.75	65.84	14.29	34.27	3.95	/
	TT	17.05	4.51	26.44	80.52	8.41	66.51	12.96	26.94	/	/
SNP 8	CC	**17.82 ^A^**	4.17	23.41	88.47	9.91	66.82	13.93	33.14	3.28	33.07
	CT	**17.69 ^AB^**	4.12	23.33	88.15	9.97	67.17	13.91	33.25	3.34	33.18
	TT	**17.44 ^B^**	4.10	23.53	87.49	9.88	67.09	14.10	33.01	3.21	32.41
SNP 9	AA	17.82	4.22	23.70	94.84	**10.67 ^a^**	66.61	14.71	34.82	3.53	34.34
	AG	17.73	4.11	23.19	90.05	**10.09 ^a^**	67.33	13.75	33.46	3.30	32.61
	GG	17.68	4.15	23.49	88.19	**9.90 ^b^**	66.98	13.88	32.93	3.23	32.56
SNP 10	CC	17.63	4.03	22.83	88.74	**9.79 ^ab^**	66.54	13.56	**31.58 ^a^**	3.19	34.46
	TC	17.62	4.13	23.43	87.34	**9.79 ^a^**	66.88	14.01	**33.20 ^b^**	3.30	33.18
	TT	17.75	4.15	23.39	88.67	**9.94 ^b^**	66.81	13.87	**33.21 ^b^**	3.26	32.74
SNP 11	AA	**17.75 ^ab^**	4.16	23.46	89.02	**10.00 ^a^**	66.52	13.99	33.29	3.28	32.76
	GA	**17.82 ^a^**	4.15	23.30	88.42	**9.88 ^ab^**	66.84	13.88	33.00	3.25	32.86
	GG	**17.57 ^b^**	4.12	23.45	87.63	**9.83 ^b^**	66.96	13.91	33.20	3.28	32.98

Note: Values with different lowercase or uppercase letters within the tag mean significant difference at *p* < 0.05 or *p* < 0.01, respectively. The same letters imply no difference (*p* > 0.05). Significance is indicated by bold type.

## Data Availability

The original contributions presented in this study are included in the article. Further inquiries can be directed to the corresponding author.

## Abbreviations

The following abbreviations are used in this manuscript:

MFD	Mean Fiber Diameter
FDSD	Fiber Diameter Standard Deviation
CVFD	Fiber Diameter Coefficient of Variation
SL	Staple Length
FC	Fineness Count
HL	Hair Length
CN	Crimp Number
GFW	Greasy Fleece Weight
LWBS	Live Weight Before Shearing
LWAS	Live Weight After Shearing
MAF	Minor Allele Frequencies
FW	Fine Wool Fiber
UFW	Ultrafine Wool Fiber
